# Comparison of the long-term cause of failure and survivorship of four hundred and twenty seven metal-on-metal hip arthroplasties: resurfacing versus large head total hip arthroplasty

**DOI:** 10.1007/s00264-021-05044-y

**Published:** 2021-06-21

**Authors:** Michele Palazzuolo, Alexander Antoniadis, Leilani Delaune, Inès Tornare, Julien Wegrzyn

**Affiliations:** Department of Orthopedic Surgery, Lausanne University Hospital – CHUV, Avenue Pierre-Decker, 4, CH-1011 Lausanne, Switzerland

**Keywords:** Metal-on-metal bearing, Total hip arthroplasty, Hip resurfacing, Complication, Survivorship, Trunnionosis

## Abstract

**Introduction:**

Comparison of mid- to long-term cause of failure and survivorship of metal-on-metal (MoM) resurfacing hip arthroplasty (RHA) and large head total hip arthroplasty (THA) remains sparse. This study aimed to identify and compare the cause of failure and survivorship of MoM RHA and THA at a minimum ten year follow-up.

**Methods:**

Four hundred twenty-seven MoM hip arthroplasties (286 THA and 141 RHA) were retrospectively analyzed at a mean follow-up of 13 ± three years. Causes of failure were reported as MoM specific (i.e., adverse reaction to metal debris (ARMD) and painful hip with ion elevation) or MoM non-specific (i.e., fracture, infection, and dislocation). Chromium (Cr) and cobalt (Co) ion levels and Co/Cr ratio were compared. Survivorship was compared according to the cause of failure with revision as the endpoint.

**Results:**

The rate of ARMD was significantly higher in THA (OR = 2.9 [95%-CI: 1–7]; *p* = 0.02). No significant difference was detected in failure rate due to other causes between the two groups (*p* = 0.2–0.9). Ion levels and Co/Cr ratio were both significantly higher in THA (*p* < 0.01). Survivorship was significantly lower in THA compared to RHA at ten years [89% (95%-CI: 85%–91%) vs 96% (95%-CI: 91%–98%); *p* = 0.01] and 15 years [73% (95%-CI: 67%–78%) vs 83% (95%-CI: 73%–90%); *p* = 0.01].

**Conclusion:**

RHA survivorship was significantly higher at any time point. Failure rate due to ARMD was significantly higher in THA while no significant difference in other causes of failure was observed between the two groups. This result emphasizes the role of fretting corrosion at the head-neck junction (i.e., trunnionosis) with significantly higher ion levels and Co/Cr ratio dissociation in THA.

## Introduction

Large head metal-on-metal (MoM) total hip arthroplasty (THA) regained popularity in the last decade of the twentieth century after the introduction of modern resurfacing hip arthroplasty (RHA). MoM bearing surfaces were postulated at that time to decrease the risk of aseptic loosening related to wear and therefore to increase implant survivorship compared to conventional metal or ceramic on polyethylene bearings [1]. However, several reports in the literature raised safety concerns about MoM bearing due to increased chromium (Cr) and cobalt (Co) blood ion concentrations [2–5], adverse reaction to metal debris (ARMD) [6–8], osteolysis [9], and implant loosening [10–13], leading to restriction of use imposed by regulatory agencies worldwide. To date, the outcome of MoM THA is known to be poorer than conventional bearings with complication rates as high as 15.5% at ten years [14, 15]. Conversely, the outcome of RHA was reported to be more favourable at mid- to long-term follow-up with survivorship ranging from 91 to 99.7% at ten years [16, 17]. Therefore, such differences in survivorship have raised questions about different modes of failure between these two MoM hip arthroplasties that could explain better long-term survivorship achieved with RHA.

However, clinical series comparing the cause of failure and survivorship between MoM THA and RHA at mid-term follow-up remain sparse [18–20] and are even lacking with consistent long-term follow-up. To our knowledge, only the study of Ridon et al. [15] compared the mid-term outcome of the same MoM bearing between THA and RHA. This study suggested the role of trunnionosis to explain the higher ion elevation and rate of failure due to ARMD in MoM THA compared to RHA [15]. However, this study included a limited number of patients with a mean follow-up of less than 10 years. Therefore, the current single-center and retrospective study on prospectively collected data aimed to identify and compare the failure mode and survivorship of MoM THA and RHA at a minimum ten year follow-up with a particular attention to specific complications related to the MoM bearing.

## Patients and methods

### Patients and procedures

**Fig. 1:**
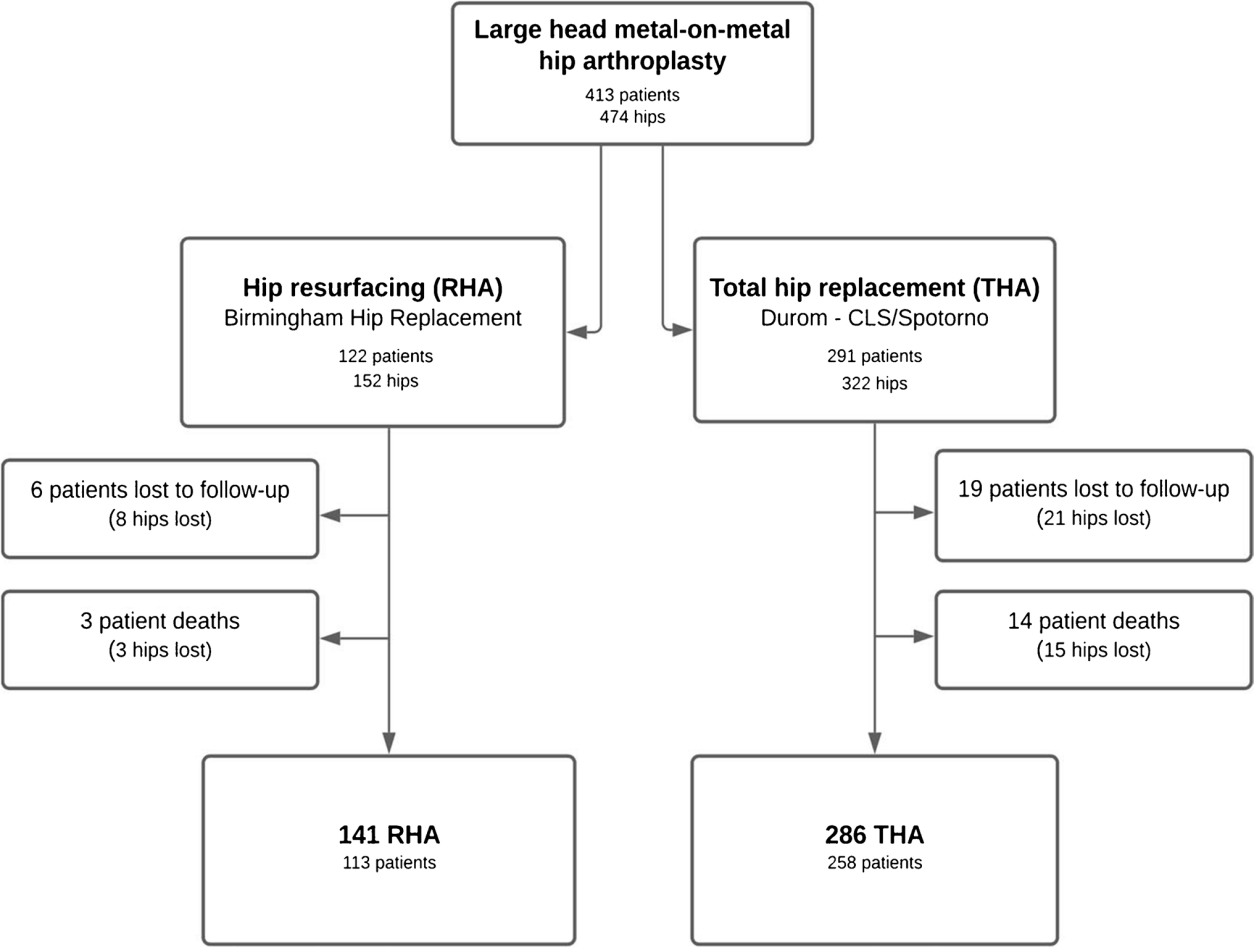
Study flowchart

Between 1998 and 2010, a continuous series of 474 MoM hip arthroplasties (413 patients, 322 THA and 152 RHA) were prospectively included in our institutional total joint registry and retrospectively analyzed at the latest follow-up (Fig. 1). The patient’s informed consent and Institutional Review Board approval were obtained before initiating this study (CER-VD #2019–02,172). All the procedures were performed in patients < 75 years with advanced hip OA excluding inflammatory, traumatic, oncologic, and septic indications. The other exclusion criteria were known allergy to metal, kidney failure, and women with childbearing potential. The implant used was Birmingham Hip Resurfacing® (Smith & Nephew, London, UK) for RHA and Durom® cup construct with Metasul® Large Diameter Head and CLS/Spotorno® stem (Zimmer, Warsaw, IN, USA) for THA. The median head diameter for THA was 46 ± 4 mm and 48 ± 4 mm for RHA. All the procedures were performed through a conventional posterolateral approach by or under the direct supervision of a single senior fellowship-trained hip arthroplasty surgeon at our institution.

At the latest follow-up evaluation, six patients (5%) in the RHA group and 19 patients (7%) were lost to follow-up. In addition, three patients (2%) in the RHA group and 19 patients (5%) in the THA group were deceased (Fig. 1). Therefore, 427 MoM hip arthroplasties including 141 RHA and 286 THA were analyzed at a mean follow-up of 13 ± three years (range: 10 to 19 years). There were 241 women (65%) and 130 men (35%) with a mean age at surgery of 55 ± 13 years for THA and of 51 ±  nine years for RHA (*p* = 0.01).

### Evaluation

Patients returned for post-operative follow-up visits at three months, six months, one year, and annually thereafter. Patients underwent an annual clinical examination, and plain antero-posterior and lateral radiographs of the pelvis and the operated-on hip were obtained. Blood ion measurements were performed. Patients who did not attend the annual visit were contacted by phone. Cobalt (Co) and chromium (Cr) ion blood levels were measured according to international standards [21]. All the patients with ion values above 119 nmol/l for cobalt and 135 nmol/l for chromium and/or painful hip underwent metal artifact reduction sequence magnetic resonance imaging (MARS-MRI) to evaluate potential adverse reaction to metal debris (ARMD) to the hip [22].

At the latest follow-up, the data from our institutional joint registry were analyzed to identify the cases which underwent revision surgery. Then, the causes of failure in the MoM RHA and THA groups were analyzed through retrospective chart review and reported as MoM specific (i.e., ARMD and painful hip with ion elevation) or MoM non-specific (i.e., periprosthetic fracture, infection, dislocation). In accordance with the recommendations of Swiss Orthopaedics [23], patients with painful hip associated with blood ion levels above 340 nmol/l for cobalt and 386 nmol/l for chromium underwent revision even in the absence of ARMD on the MARS-MRI evaluation. The revision was defined as the exchange of either one of the components or all of them. Revision for any reason was considered as the endpoint for survivorship analysis.

### Statistical analysis

An a priori power analysis was performed to confirm that this study is adequately powered. Indeed, the total sample size to be included was 280 patients to detect a significant difference in survivorship between the two groups with a significance level of 0.05 and a power of 0.8.

Quantitative variables are presented as mean ± standard deviation. Comparison of continuous and quantitative variables between the two groups was performed using two-sample *t*-tests. Comparison of qualitative variables between the two groups was performed using Fisher’s exact tests. Survivorship analyses were performed using Kaplan–Meier curves with 95% confidence intervals (95%-CI) at 5, 10, and 15 years using RHA or THA revision for any cause as the endpoint. Comparative survivorship analyses between the two groups were performed according to the cause of failure (i.e., MoM specific or non-specific) using log-rank tests. A linear regression model was applied to detect a possible correlation between head diameter and specific metal-on-metal failure causes for both THA and RHA. Statistical analyses were performed using the SPSS version 22 software (SPSS Inc, Chicago, IL) with a level of significance set at *p* < 0.05.

## Results

### Modes of failure

At a mean follow-up of 13 ± three years, the overall rate of failure was 20% (56/286 hips) in THA and 11% (16/141 hips) in RHA (*p* = 0.03) (Table 1).

**Table 1:** Causes of failure of metal-on-metal (MoM) resurfacing (RHA) and large head total hip arthroplasty (THA)

	**THA**	**RHA**	
	**N (%)**	**N (%)**	**p-value**
**MoM specific**			
**ARMD**	33(59)	6(38)	0.02
**Pain**	12(21)	7(44)	0.80
**MoM non-specific**			
**Fracture**	3(5)	2(12)	0.67
**Infection**	6(11)	0 (0)	0.18
**Instability**	2 (4)	1(6)	>0.99
**Total**	56	16	0.03

#### MoM specific

The most common cause of failure of THA was ARMD (33/56 THA, 59%), whereas the main cause of failure of RHA was painful hip with ion elevation (7/16 RHA, 44%) (Table 1). Failure due to ARMD was significantly higher in THA than in RHA (33 THA [59%] vs 6 RHA [38%], *p* = 0.02; OR 2.93, 95%-CI: 1.2–7.2). No significant difference was detected in the failure rate due to painful hip with ion elevation (*p* = 0.8) (Table 1). In addition, no significant correlation was detected between femoral head size and MoM-specific modes of failure in a linear regression model for both THA and RHA (*R*^2^ = 0.098 and *R*^2^ = 0.094, respectively).

#### MoM non-specific

The most common cause of failure of large head MoM THA was infection (6/56 THA, 11%), while the main cause of failure of MoM RHA was periprosthetic femoral neck fracture (2/16 RHA, 12%) (Table 1). No significant difference was detected in the failure rate due to MoM non-specific causes between the two groups (*p* = 0.2 to 0.9, Table 1).

### Co and Cr ions

**Fig. 2:**
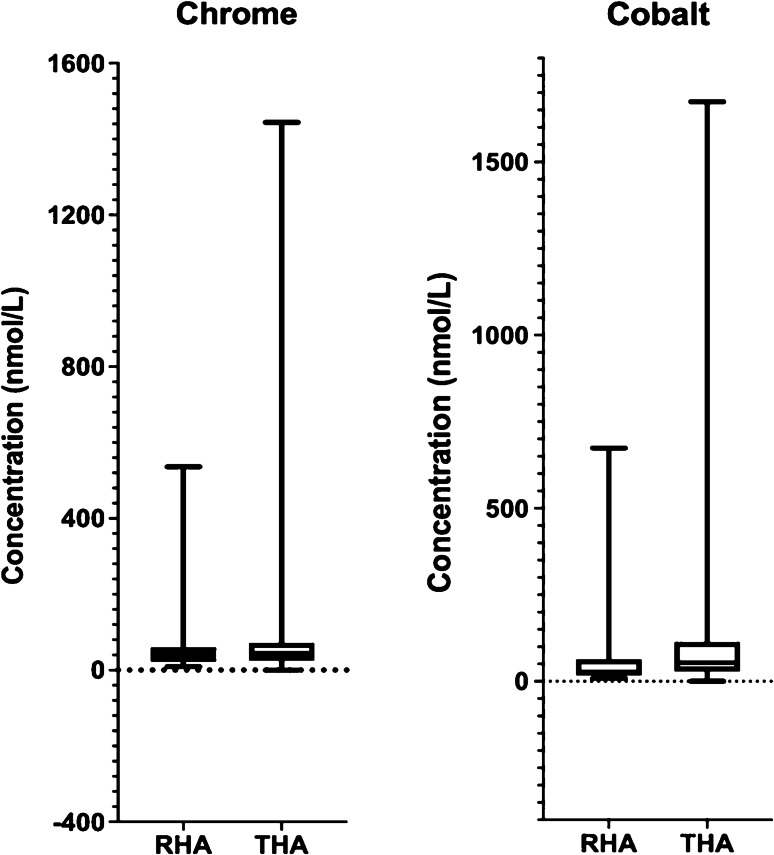
Chrome and cobalt ion level in metal-on-metal resurfacing (RHA) and large head total hip arthroplasty (THA)

**Fig. 3:**
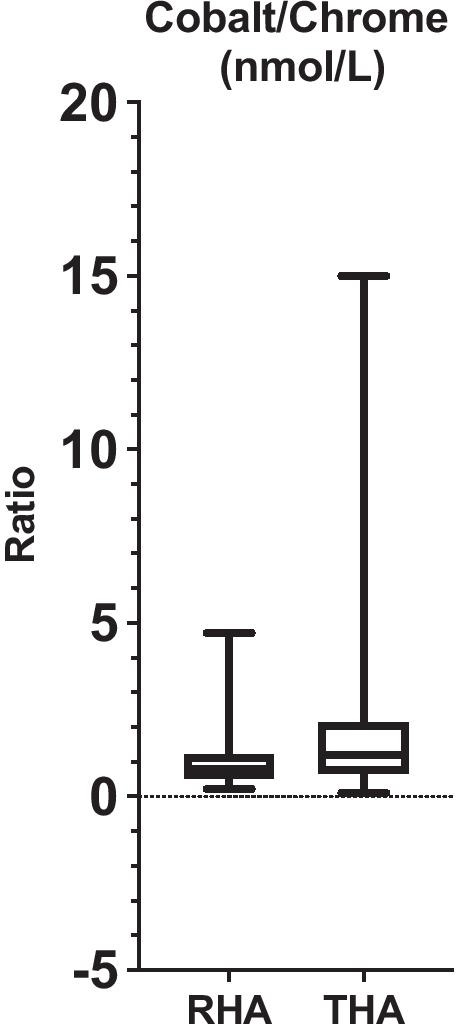
Cobalt/chrome ratio in metal-on-metal resurfacing (RHA) and large head total hip arthroplasty (THA)

At the latest follow-up, the Co level was significantly higher in THA compared to RHA (53 ± 94 nmol/L vs 26 ± 78 nmol/L; *p* < 0.001) (Fig. 2). No significant difference was detected in Cr level between THA and RHA (44 ± 73 nmol/L vs 41 ± 56 nmol/L; *p* = 0.3) (Fig. 2). The Co/Cr ratio was significantly higher in THA compared to RHA (1.78 ± 1.82 vs 0.94 ± 0.63; *p* < 0.0001) (Fig. 3).

### Survivorship

**Fig. 4:**
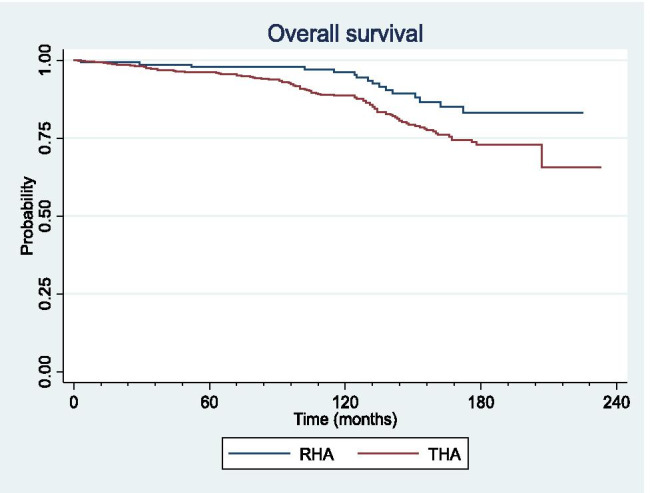
Overall survivorship curves for metal-on-metal resurfacing (RHA) and large head total hip arthroplasty (THA)

Overall survivorship was significantly higher in RHA compared to THA at 5 years (98% [95%-CI: 94%–99%] vs 96% [95%-CI: 94%–98%]; *p* = 0.01), ten years (96% [95%-CI: 91%–98%] vs 89% [95%-CI: 85%–91%]; *p* = 0.01), and 15 years (83% [95%-CI: 73%–90%] vs 73% [95%-CI: 67%–78%]; *p* = 0.01) (Fig. 4).

**Fig. 5:**
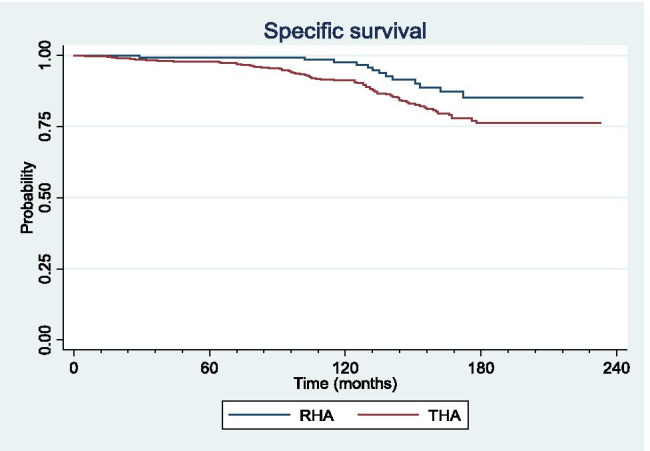
Survivorship curves for metal-on-metal specific cause of failure for resurfacing (RHA) and large head total hip arthroplasty (THA)

Survivorship related to MoM-specific causes of failure was significantly higher in RHA compared to THA at five years (99% [CI 95%–99%] vs 98% [CI 96%–99%]; *p* = 0.02), ten years (98% [CI 93%–99%] vs 90% [CI 88%–93%]; *p* = 0.02), and 15 years (85% [CI 75%–91%] vs 76% [CI 70%–80%]; *p* = 0.02) (Fig. 5).

**Fig. 6:**
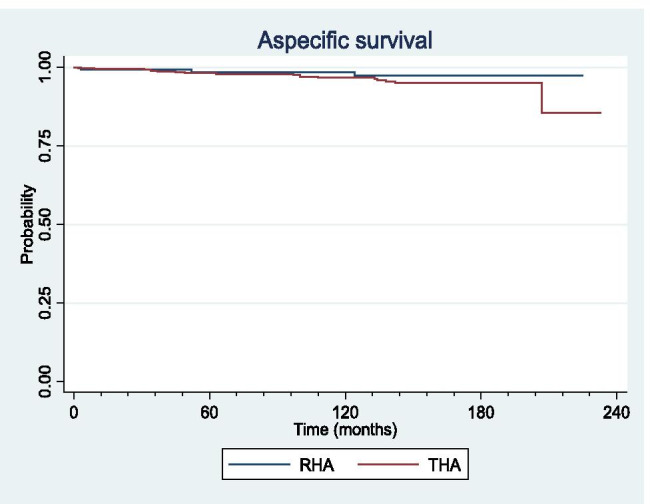
Survivorship curves for metal-on-metal non-specific cause of failure for resurfacing (RHA) and large head total hip arthroplasty (THA)

No significant difference was detected in survivorship related to MoM non-specific causes of failure between the two groups at five, ten or 15 years (*p* = 0.3) (Fig. 6).

## Discussion

MoM bearing remains controversial in RHA and has been largely abandoned in THA due to early catastrophic failures such as ARMD associated with potential major bone loss and severe damages to the soft tissue at the time of revision [24, 25]. Conversely, RHA is still performed in some centers and reported with excellent functional outcome and survivorship up to 99.7% at 10 years [16, 26]. However, series that compared survivorship of MoM RHA and large head THA with consistent long-term follow-up are lacking. Therefore, this study aimed to identify and compare the mode of failure and survivorship of RHA and THA at a minimum ten year follow-up with a particular attention to specific complications related to MoM bearing. The most important finding of the present study was that survivorship of RHA was significantly higher than survivorship of large head MoM THA at five, ten and 15 years after surgery. Importantly, this difference in survivorship was explained by a 2.93-fold increase in failure rate due to ARMD in THA. Indeed, ion levels and dissociation of Co/Cr ratio were significantly higher in THA. Therefore, these results emphasize the potential role of trunnionosis as the main mechanism of failure in THA and not only the MoM bearing by itself.

The overall 15-year survivorship of 83% in RHA and 73% in THA observed in the current study was in agreement with the previous series [15, 27–35]. Indeed, Ng et al. and Althuizen et al. reported a high failure rate for Durom® MoM THA with a ten year revision rate ranging from 14 to 31% [36, 37]. In our series, Co level and Co/Cr ratio were significantly higher in THA compared to RHA. These findings suggest an additional source of ion production in THA other than the head-cup bearing interface [4, 38, 39]. A similar result was described by Ridon et al. and Johnson et al. in series comparing RHA and THA constructs performed with the same acetabular component [15, 40]. In addition, Garbuz et al. showed that patients with a large head MoM THA presented with a 46-fold and tenfold increase in Co and Cr levels respectively compared to RHA [18]. Goldberg et al. demonstrated that Cr release remains localized around the taper of the femoral stem, while Co is released into the blood, leading to a higher blood level of Co and dissociation of the Co/Cr ratio in THA [38]. Therefore, this Co/Cr ratio dissociation could be supposed to be a direct consequence of fretting corrosion (i.e., trunnionosis) at the head-neck junction. Moreover, trunnionosis was also described as the result of a local interplay between the head/taper engagement levels, and the horizontal lever arm and load offset applied on the trunnion [41–43]. Consequently, the contribution of trunnionosis to metal ion production and ARMD could be considered as a natural phenomenon in large head MoM THA that is not influenced by implant positioning or edge loading at the MoM bearing level [43]. Therefore, we believe that ion production at the Morse taper interface could explain the significantly higher incidence of ARMD and lower implant survivorship in the THA group of the current study. In addition, the metal debris production due to trunnionosis might increase third body wear at the MoM bearing interface and therefore increase the risk of ARMD in THA [43]. By definition, RHA is not affected by potential trunnionosis. This could explain the significantly lower ion levels and ARMD rate, and higher survivorship compared to THA we observed in the current long-term follow-up study. Taking altogether, these results suggest that RHA may be a valid bone preserving option in carefully selected patients [16]. Our study presented with some limitations. First, this total joint registry study was observational and focused on revision rate and survivorship. This study did not aim to evaluate functional or radiological outcomes. Second, the indication for a RHA or THA procedure was at the senior surgeon’s discretion, with RHA mainly performed in younger and more active patients presenting with primary hip osteoarthritis. Therefore, the two groups were not matched for age, sex, patient’s functional demand, or indication. Third, no analysis of the implants was performed after RHA or THA revision. Especially, no trunnion analysis such as tribo-corrosion was performed on the explanted femoral stem neck and head. Only gross macroscopic assessment of the trunnion was mentioned in operative reports.

## Conclusion

Survivorship of MoM hip arthroplasty was significantly higher in RHA than in THA at any time point. Failure rate due to ARMD was significantly higher in THA, while no significant difference in the other causes of failure was observed between the two groups. Along with significantly higher ion levels and Co/Cr ratio dissociation, this result emphasized the potential role of fretting corrosion at the head-neck junction in THA. Therefore, aside from the MoM bearing itself, trunnionosis might significantly contribute to the higher rate of ARMD and poorer survivorship in THA compared to RHA.

## Data Availability

All the data and material are saved in an anonymized repository file folder and available upon request.
